# Leveraging avidin-biotin interaction to quantify permeability property of microvessels-on-a-chip networks

**DOI:** 10.1152/ajpheart.00478.2021

**Published:** 2021-11-12

**Authors:** Feng Gao, Haoyu Sun, Xiang Li, Pingnian He

**Affiliations:** Department of Cellular and Molecular Physiology, College of Medicine, Penn State University, Hershey, Pennsylvania

**Keywords:** application of avidin-biotin interaction, glycocalyx, microvessel permeability, microvessels-on-a-chip, solute transport pathways

## Abstract

Microvessels-on-a-chip have enabled in vitro studies to closely simulate in vivo microvessel environment. However, assessing microvessel permeability, a functional measure of microvascular exchange, has not been attainable in nonpermeable microfluidic platforms. This study developed a new approach that enables permeability coefficients (*P*_s_) to be quantified in microvessels developed in nonpermeable chip platforms by integrating avidin-biotin technology. Microvessels were developed on biotinylated fibronectin-coated microfluidic channels. Solute transport was assessed by perfusing microvessels with fluorescence-labeled avidin. Avidin molecules that crossed endothelium were captured by substrate biotin and recorded with real-time confocal images. The *P*_s_ was derived from the rate of avidin-biotin accumulation at the substrate relative to solute concentration difference across microvessel wall. Avidin tracers with different physiochemical properties were used to characterize the barrier properties of the microvessel wall. The measured baseline *P*_s_ and inflammatory mediator-induced increases in *P*_s_ and endothelial cell (EC) [Ca^2+^]_i_ resembled those observed in intact microvessels. Importantly, the spatial accumulation of avidin-biotin at substrate defines the transport pathways. Glycocalyx layer is well formed on endothelium and its degradation increased transcellular transport without affecting EC junctions. This study demonstrated that in vitro microvessels developed in this simply designed microfluidics structurally possess in vivo-like glycocalyx layer and EC junctions and functionally recapitulate basal barrier properties and stimuli-induced responses observed in intact microvessels. This new approach overcomes the limitations of nonpermeable microfluidics and provides an easily executed highly reproducible in vitro microvessel model with in vivo microvessel functionality, suitable for a wide range of applications in blood and vascular research and drug development.

**NEW & NOTEWORTHY** Our study developed a novel method that allows permeability coefficient to be measured in microvessels developed in nonpermeable microfluidic platforms using avidin-biotin technology. It overcomes the major limitation of nonpermeable microfluidic system and provides a simply designed easily executed and highly reproducible in vitro microvessel model with permeability accessibility. This model with in vivo-like endothelial junctions, glycocalyx, and permeability properties advances microfluidics in microvascular research, suitable for a wide range of biomedical and clinical applications.

Listen to this article's corresponding podcast at https://ajpheart.podbean.com/e/new-permeability-assessment-of-microvessels-on-a-chip/.

## INTRODUCTION

Microvessels are the site for fluid and solutes exchange between blood and surrounding tissues and are essential to the goal of circulation. The permeability properties of microvascular walls determine the selectivity of all pathways across microvessel wall for fluid and solutes transport and are critical for the exchange function. Clinical and experimental evidence indicate that increased vascular permeability results in tissue edema, driving the deleterious consequences of many disease-associated vascular complications. It is thus important to assess permeability properties of the microvessels under investigation to identify critical factors that initiate permeability increases and contribute to the progression of microvascular barrier dysfunction. Such information is crucial to a better understanding of the pathogenesis of cardiovascular diseases and to the development of novel therapeutic approaches.

Permeability measurements have been used for the assessment of microvessel barrier function in vivo or ex vivo in whole animal, organ, and individually perfused microvessels, as well as in vitro using cultured microvascular endothelial cells (ECs) (1, 2). Based on current literature information, the baseline permeability of cultured EC monolayers typically is orders of magnitude higher than those measured in intact microvessels, which manifests a proinflammatory phenotype (2). Recent advancements in microfluidic technologies have enabled in vitro cell culture conditions to closely simulate the in vivo tissue environment (3). ECs grown in microfluidics with continuous flow have shown in vivo-like endothelial junctions and surface layer of glycocalyx (4–7). The engineered vascular networks have demonstrated comparable microvascular structures and stimuli-induced cellular responses to those observed in intact microvessels (3–11). Increasing evidence indicates that cultured microvascular networks have great potentials for a broad range of human disease-related studies, such as angiogenesis, thrombosis, hemodynamic impact on ECs, blood cell/EC interactions, tumor metastases, and stimuli-induced EC [Ca^2+^]_i_ and nitric oxide signals (4–9). However, acquiring quantitative assessment of microvessel permeability, an important functional measure of microvessels, has not been attainable in all chip platforms. The commonly used polydimethysiloxane (PDMS)-based microfluidic devices in the absence of hydrogel implantation are impermeable for solute transport and, therefore, are not suitable for permeability measurements. Permeability assessments often require more complex microfluidic designs, such as diffusive hydrogel-based platforms, two-layer membrane-based microfluidics, or self-assembled vascular networks within hydrogel matrices (7, 10–17). In addition, the variable diffusive property of each matrix composition requires detailed characterizations of solute transfer within each microfluidic scaffold to achieve a standardized quantification of microvessel permeability (8, 13, 18). These complexities have thus prevented the quantitative assessments of permeability as a standard functional measure of microvessels in many chip platforms.

Recently, avidin-biotin interactions have been used in static cultured EC monolayers to illustrate local leakages and transport pathways (19, 20). In this study, we applied the avidin-biotin technology to microfluidic microvascular networks and provide an easily implemented method to quantify permeability coefficients in microvessels grown in PDMS-based devices. Using this approach, we delineated the temporal and spatial changes in permeability coefficients to solutes with different physicochemical properties and transport pathways under both control and stimulated conditions. The importance of assessing permeability coefficients such as *P*_s_ over commonly used fluorescence intensity measurements of transported molecules (19–21) is that permeability coefficients incorporate the driving force for solute and fluid transport, vessel geometry, and the surface area for transport into the equation, representing the true permeability properties of the vascular wall, and therefore could be used to compare permeability properties of the vascular wall between studies or cross platforms. The quantitative measures of permeability coefficients presented here enable the barrier function of microvessels developed in different chip platforms to be compared with those measured in intact microvessels and, therefore, further validate their utilities for biomedical applications.

## MATERIALS AND METHODS

### Microfluidic Device Design and Fabrication

**Figure 1.**
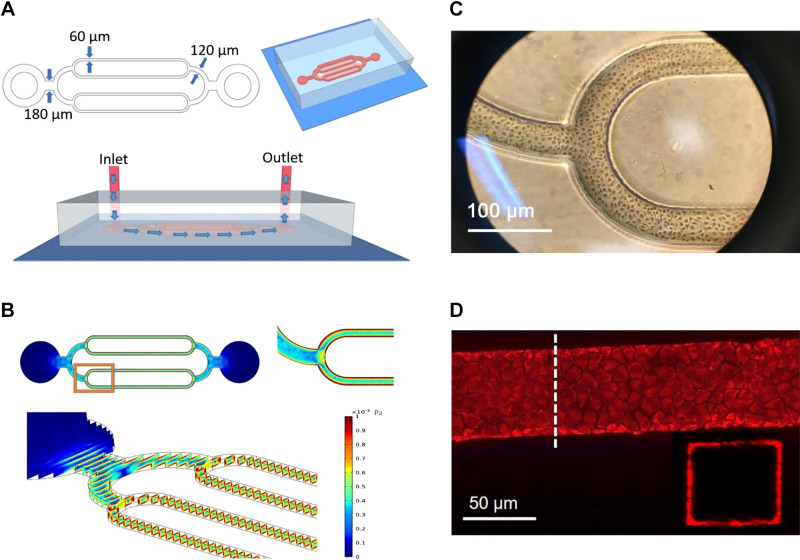
Schematic illustration of the microfluidics and sheer stress distribution within microchannel networks. *A*: microfluidic pattern, channel diameters, and perfusion illustration. *B*: simulation of wall shear stress distribution within the microchannel networks via COMSOL Multiphysics modeling software, which was based on device geometry, a 0.22 µL/min perfusion rate, and a culture medium viscosity at 37°C. *C*: endothelial cells under culture in microchannels (bright field). *D*: confocal images of the vascular wall endothelial cells outlined by Calcein Red at *X–Y* and *X–Z* focal planes of the microchannels.

The microchannel pattern is designed as a two-level branched microchannel network with channel widths at 120 µm and 60 µm and a uniform height of 80 µm. Standard photolithography was used for the master mold fabrication and PDMS soft lithography was used for the microfluidic microchannel network fabrication. The base was formed by a glass coverslip (No. 1) spin-coated with a thin layer of PDMS to permit high-resolution imaging. At the distal end of each mother channel, an inlet and an outlet were created with a biopsy punch for cell loading, media, reagent perfusion, and perfusate collection. The schematic design of the device is shown in Fig. 1*A.* The shear stress distribution within the microchannel network was simulated by COMSOL Multiphysics modeling software and illustrated in Fig. 1*B.* Calculations were based on device geometry, a 0.22 µL/min perfusion rate and a culture medium viscosity at 37°C. Details have been previously described (4, 6).

### In Vitro Microvessel Development in Microchannel Networks

Each device was first loaded with deionized water and sterilized with UV light for 8 h. After sterilization, microchannels were rinsed with phosphate-buffered saline (PBS) and coated with fibronectin solution (100 μg/mL, Cat. No. PHE0023, Gibco) containing biotin (0.25 mg/mL, Cat. No. 21338, Thermo Fisher Scientific) overnight at 4°C. The device was then rinsed with PBS to remove the free coating solution and reloaded with culture medium followed by a 15-min incubation at 37°C before cell seeding. Primary rat dermal microvascular endothelial cells (RDMECs) and culture medium were purchased from Cell Biologics (Cat. No. M1266 and RA-6064). Cultured RDMECs between *passages 2* and *5* were used for this study. RDMECs in suspension were loaded into channels from the device inlet and cultured in 5% CO_2_ incubator at 37°C without perfusion for ∼5 to 6 h to allow cell adhesion to the coating layer along the microchannels. Additional cell loading and gentle device titling were used to achieve uniform cell adhesion at the desired cell density. After cell seeding, culture medium was perfused into the microchannels at a constant flow rate of 0.22 μL/min through an inlet tubing connected to a motor pump (Harvard Apparatus Holliston, MA). After 2 to 3 days of culture, the in vitro microvessels were well formed and ready for experiments (Fig. 1*C*). Details have been previously described (4).

### Confocal Fluorescence Imaging

Leica TCS SP8 confocal system with solid-state lasers and hybrid detectors (HyDs) were used for fluorescence imaging. Leica ×40 objective (NA 1.3) was used for image acquisitions except where noted otherwise. The excitation and emission laser wavelength settings used for each fluorescent tracer are listed in appendix Table A1.

For immunofluorescent staining of vascular endothelial (VE)-cadherin, RDMECs were fixed with 2% paraformaldehyde and blocked with 1% bovine serum, followed by 0.1% Triton X-100 for permeabilization before staining for VE-cadherin (primary antibody, Cat. No. SC-6458, Santa Cruz; secondary antibody, Cat. No. A-11055, donkey-anti-goat IgG, Life Technologies). Fluorescence images were obtained after nuclei staining with DAPI for 10 min (300 nM).

Fluo-4 AM was used to measure EC intracellular calcium concentration ([Ca^2+^]_i_). Devices with well-developed microvessel networks were first perfused with Fluo-4 AM (5 µM, Cat. No. F23917, Life Technologies) in 1% BSA-Ringer solution for 40 min, followed by BSA-Ringer perfusion alone for 5 min to wash the free Fluo-4 AM from the vessel lumen before collecting baseline images. Platelet-activating factor (PAF; 10 nM, Sigma) that has known effects on EC [Ca^2+^]_i_ and microvessel permeability (22) was selected as a representative agonist to induce increases in EC [Ca^2+^]_i_ and permeability in RDMEC-formed microvessels. Calcium and permeability images were recorded simultaneously for this set of experiment.

Visualization of glycocalyx on ECs was achieved by FITC-lectin staining (5 µM, Cat. No. F14201, Life Technologies). Calcein Red (3 µM) and DAPI (300 nM) were used to outline ECs and nuclei, respectively. Images were collected before and after perfusion with either 10 nM PAF or 10 µM hydrogen peroxide for up to 30 min. The images of calcein-labeled vascular wall ECs at *X–Z* focal planes are shown in Fig. 1*D.* The cross-sectional image at *X–Z* focal plane was acquired using the super Z-galvanometric stage. The thickness of glycocalyx was quantified by measuring the height (number of pixels) of FITC-lectin-labeled glycocalyx above Calcein Red-labeled endothelial cells on the *X–Z* cross-sectional confocal images and converted to the length based on the system calibration at 0.042 µm/pixel. Leica SP8 confocal hybrid detector offers near zero noise. The signal-to-noise ratio of FITC-lectin is ∼15–30:1.

### Measurements of Solute Permeability Coefficient Using Biotin-Avidin Interaction in Microvessels-on-a-Chip Networks

The quantification of solute permeability coefficient (*P*_s_) was slightly modified from *P*_s_ measurements in individually perfused microvessels based on Fick’s first law where *P*_s_ is a measure of solute flux (*J*_s_) per unit area of microvessel wall (*A*) in relation to the solute concentration difference (Δ*C*) across the microvessel wall (23)

(*1*) $$P_{s}=\left(\frac{J_{s}}{A}\right)/\Delta C.$$

At the beginning of the experiment, Δ*C* is equal to the initial concentration of the solute in the vessel lumen (C_0_) due to the negligible amount of the solute at extracellular space. The value of C_0_ represents the initial number of molecules (*N*_0_) per vessel lumen volume (V_c_), C_0_ = *N*_0_/V_c_, and *J*_s_ is calculated from the rate of the molecules across the endothelium.

(*2*) $$P_{\text{s}}=\frac{1}{N_{0}}.\frac{\text{d}N}{\text{d}t}.\frac{{\text{V}}_{\text{c}}}{A}$$

**Figure 2.**
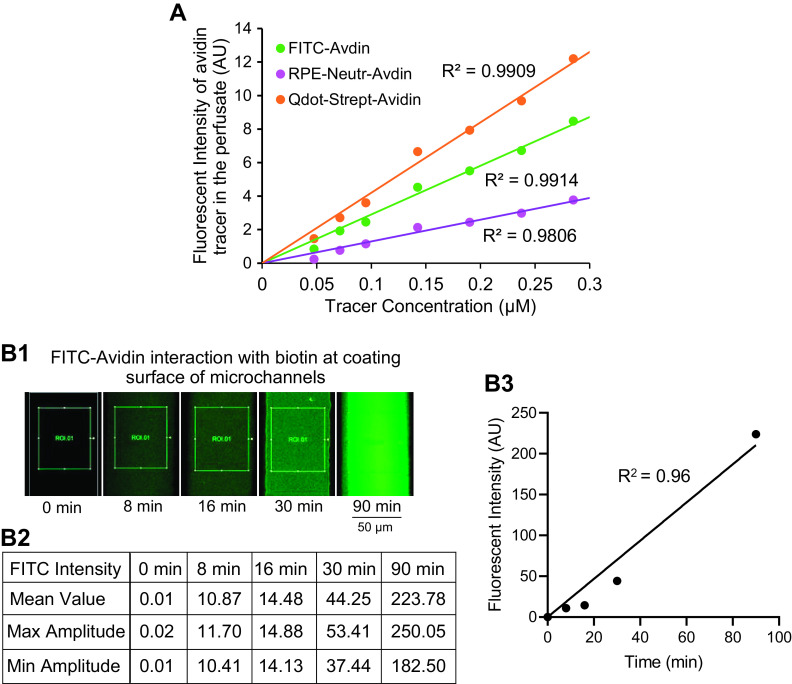
Fluorescence intensity calibration of the microfluidic device. *A*: linear relationships between the molecule fluorescence intensity and the molecule concentration. Each avidin tracer was perfused into microfluidic networks in the absence of endothelial cells (ECs) and biotin coating, and the fluorescence intensity (FI) in the vessel lumen is plotted as a function of tracer concentration. *B*: the coating layer biotin-binding capacity to FITC-Avidin. Microfluidic device coated with fibronectin solution containing biotin (0.25 mg/mL) in the absence of ECs was perfused with FITC-Avidin at the experimental concentration of 0.09 µM in 1% BSA-Ringer solution. During image collections, the channel lumen was briefly perfused with tracer-free 1% BSA-Ringer solution and resumed tracer perfusion right after. Images were collected at different FITC-Avidin perfusion time (*B1*). The recoded FI represents the FITC-Avidin captured by biotin at the coating layer and the measured FI values are shown in *B2*. The increased FI as a function of FITC-Avidin perfusion time is plotted in *B3*. The time-dependent increases in FITC-Avidin binding reached mean FI of 224 AU after 90 min of perfusion, which is far beyond the biotin-binding capacity required for our experiments. All tests presented in *A* and *B* were conducted under the same image settings as those used for experimental image collections. AU, arbitrary unit; RPE, R-Phycoerythrin.

As the fluorescence intensity (*I*) recorded in the images is in proportion to the number of solute molecules (Fig. 2*A*), *Eq. 2* is then be converted to the following:

(*3*) $$P_{\text{s}}=\frac{1}{I_{\text{0}}}.\frac{\text{d}I}{\text{d}t}.\frac{{\text{V}}_{\text{c}}}{A}.$$

For cylindrical geometry of the vessels, $\frac{{\text{V}}_{\text{c}}}{A}$ equals half the radius. Our microfluidic channels have a rectangular geometry (Fig. 1*D*) where $\frac{{\text{V}}_{\text{c}}}{A}$ is equal to $\frac{ab}{2(a+b)}$ where *a* and *b* are the width and height of the channel, respectively. *P*_s_ calculation of the microvessels in microfluidic devices was thus

(*4*) $$P_{\text{s}}=\frac{1}{I_{\text{0}}}.\frac{\text{d}I}{\text{d}t}.\frac{ab}{2(a+b)}.$$

In this study, the solute flux is the rate of avidin tracers across the endothelium. Each Avidin, Streptavidin, or NeutrAvidin protein can bind up to four biotin molecules with kinetics of 7 × 10^7^ M^−1^·s^−1^ (24, 25). This fast reaction rate suggests that the diffusion of avidin to its binding site is one of the rate-limiting factors for their interaction (24). Based on the fast-binding kinetics and high binding affinity between biotin and avidin, as well as sufficient biotin available for avidin binding (ratio of avidin to biotin at 1:10,000) under our experimental conditions, we assume that all the avidin molecules crossed ECs were immediately captured and immobilized by substrate biotin, forming stable biotin-avidin complex, and were not diffusible molecules anymore. Therefore, the rate of biotin-avidin complex formation (d*I*/d*t*, the slope of the FI profile) represents the solute flux, which is diffusion controlled, whereas the amount of the immobilized biotin-avidin accumulation at the coating layer should not affect the diffusion process. Since the PDMS channel is impermeable to both solute and liquid, there was no convective transport and the diffusible solute outside of EC layer is negligible. The binding between biotin and avidin, due to the high binding strength, is near irreversible, and the formation of biotin-avidin complex is cumulative. Based on the characterizations of biotin-avidin interaction, *P*_s_ was then calculated based on the slope of accumulated Avidin FI (d*I*/d*t*) at substrate, the tracer FI in the vessel lumen (*I*_0_) during vessel perfusion, and the vessel dimension (*Eq. 4*). The transport pathways of each tracer were differentiated by the spatial distribution of the tracers at substrate in relation to the cell structures in the vessel wall. Different size and charge of fluorescence-labeled Avidin, Neutr-Avidin, and Strept-Avidin were used for *P*_s_ measurements, which are FITC-Avidin, R-Phycoerythrin (RPE)-Neutr-Avidin, nanoparticle (Qdot 800)-Strept-Avidin, and Texas Red-Avidin. Leica TCS SP-8 confocal system and a Leica ×25 objective (NA, 0.95) were used for image collection. Each fluorescent tracer was perfused into microvessel networks at a concentration of 0.09 µM in 1% BSA-Ringer solution.

To assure sufficient binding capacity of biotin to avidin molecules crossed EC layer, the concentration ratio of avidin tracer to biotin at coating layer was ∼1:10,000 (0.09 µM:1 mM) in all experiments. The linearity of the lumen tracer fluorescence intensity (FI) as a function of tracer concentration is shown in Fig. 2*A*, which was conducted in microchannels in the absence of ECs and biotin coating. The binding capacity of biotin at the coating layer to avidin molecules was tested in FITC-Avidin (0.09 µM) perfused biotin-coated microchannels in the absence of ECs. Image settings in all tests were identical to those used in the experiment. Images shown in Fig. 2*B1* were acquired during the brief perfusion of tracer-free solution to wash away the unbound tracer in the channel lumen, and therefore, the FI recorded was only from the FITC-Avidin that was bound to biotin at coating layer (Fig. 2*, B2–B3*). Results showed that FITC-Avidin binding to biotin at coating layer continuously increased in a time-dependent manner and the FI was up to 223.78 (arbitrary units, AU) after 90 min perfusion, far beyond the maximum FI detected during the experiments. These results provide experimental evidence that the coating layer has sufficient biotin to catch all the avidin molecules crossed ECs under our experimental conditions.

In permeability studies, all images were captured in the absence of free tracer in the vessel lumen, i.e., the vessel networks were briefly perfused with tracer-free solution during image collection and resumed tracer perfusion right after. This step not only washed away residual molecules in the vessel lumen and any fluid containing free unbound avidin molecules in the channel including fluid at cell junctions to make sure the FI measured represents the amount of avidin flux captured by the substrate biotin, but also resets the concentration gradient and lumen background to their initial states for each image collected. Baseline permeability was measured during 20 min of tracer perfusion and platelet-activating factor (PAF, 10 nM) was applied after baseline measurements. Stacks of images were acquired with a 1,024 × 1,024-pixel scan format at 0.35-μm *z*-step in 2-min intervals. Mean FI was quantified from identical segments of vessels before and after stimulus application. Three consecutive focal planes from each *Z*-stack confocal images were used for FI quantification and data were derived from four segments of the microfluidic channels from each device. Each set of experiments was conducted in greater than or equal to four devices, and the *n* is the mean of each device, presented as means ± SE. To ensure robust and unbiased results for rigorous and reproducible scientific findings, cross checking results, blinded data analyses, and appropriate statistical analyses were used for data processing.

## RESULTS

### Continuous Flow in Microfluidics Facilitates the Formation of In Vivo-like Adherens Junctions between Endothelial Cells

**Figure 3.**
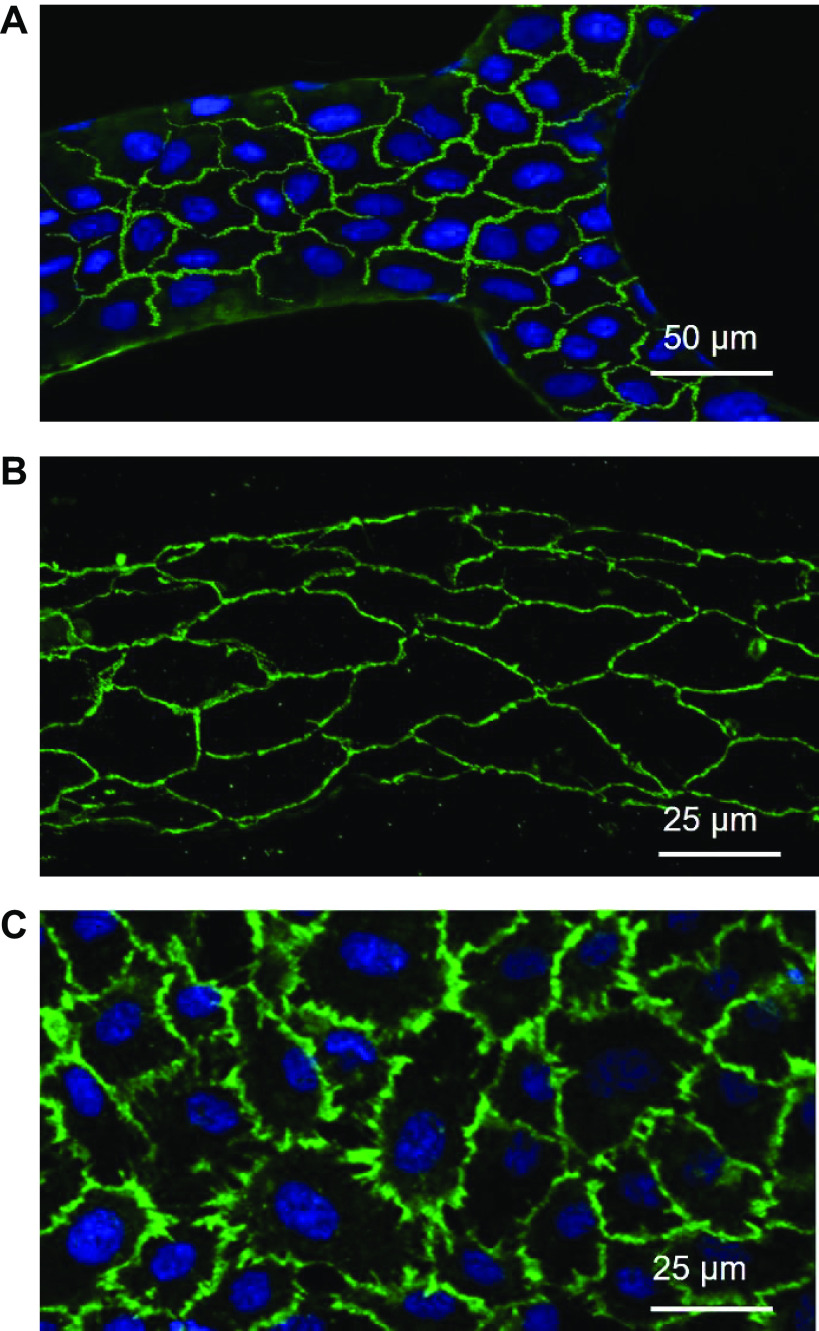
In vivo-like vascular endothelial (VE)-cadherin formation between endothelial cells (ECs) grown in microfluidic channels. *A*: VE-cadherin distribution of rat dermal microvascular ECs (RDMECs) within the perfused microchannel networks, which is similar to the pattern observed in intact rat mesenteric venule [*B*, previously published in Ref. 6 used with permission]. *C*: VE-cadherin distribution of RDMECs cultured under static conditions. VE-cadherin was conjugated with FITC (green), and cell nucleus was stained with DAPI (blue).

Junctions between ECs play important roles in regulating microvascular barrier function, and vascular endothelial (VE)-cadherin is a major adhesion molecule of adherens junctions between ECs. To evaluate the EC junctions in RDMEC-formed microvessels, VE-cadherin was immuno-labeled in microfluidic microvessels and its distribution pattern was compared with those in intact rat mesenteric venules and RDMECs grown under static culture conditions. Figure 3*A* shows well-formed VE-cadherin between RDMECs in microfluidic microvessel networks. Importantly, its smooth and continuous distribution at cell junctions reflected the characteristics of the rat venules in vivo (Fig. 3*B*) (6), which was in contrast to the stitching pattern commonly observed in ECs cultured under static conditions (Fig. 3*C*). The formation of in vivo-like adherens junctions in microvessels grown in microfluidics indicates that flow plays an important role in junctional formation between ECs and that our microfluidic cell culture conditions enabled an in vitro environment closer to the physiological in vivo setting when compared with static cell culture conditions.

### Baseline Permeability Coefficients of the In Vitro Microvessels

**Figure 4.**
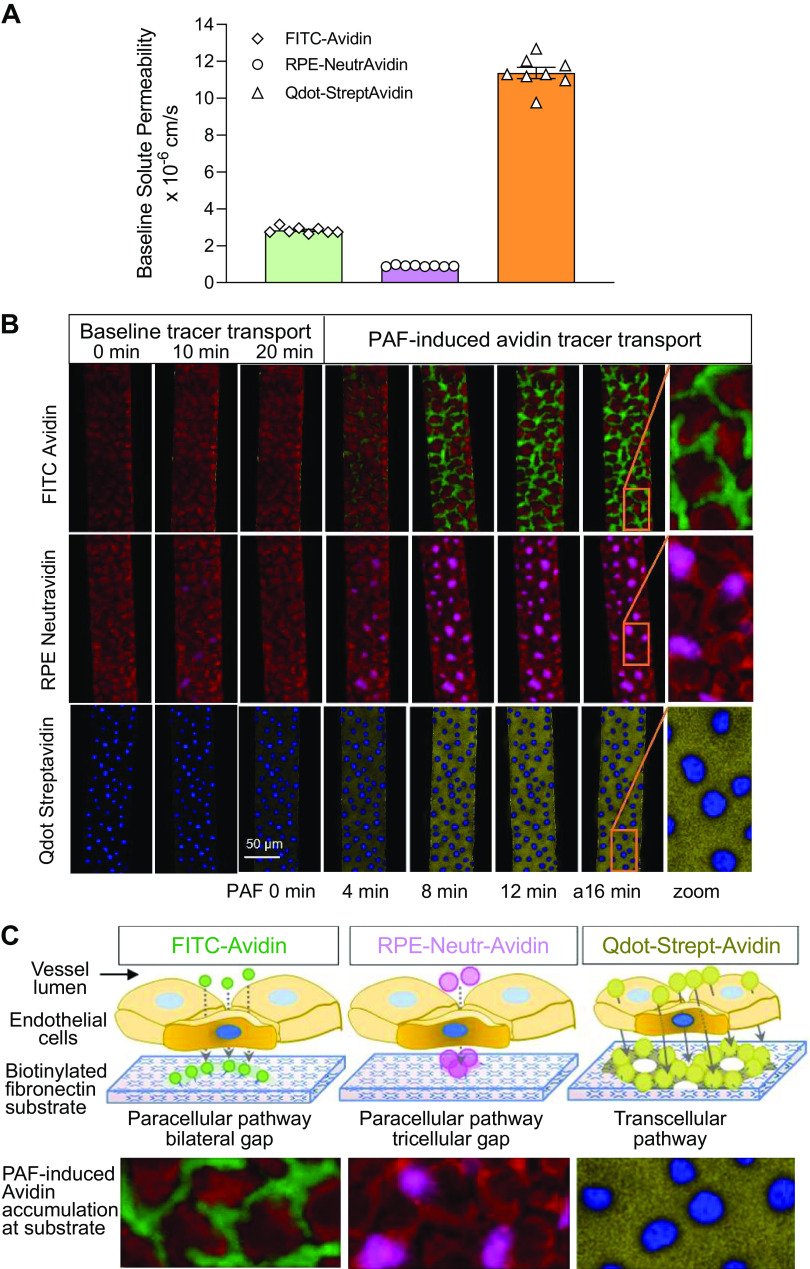

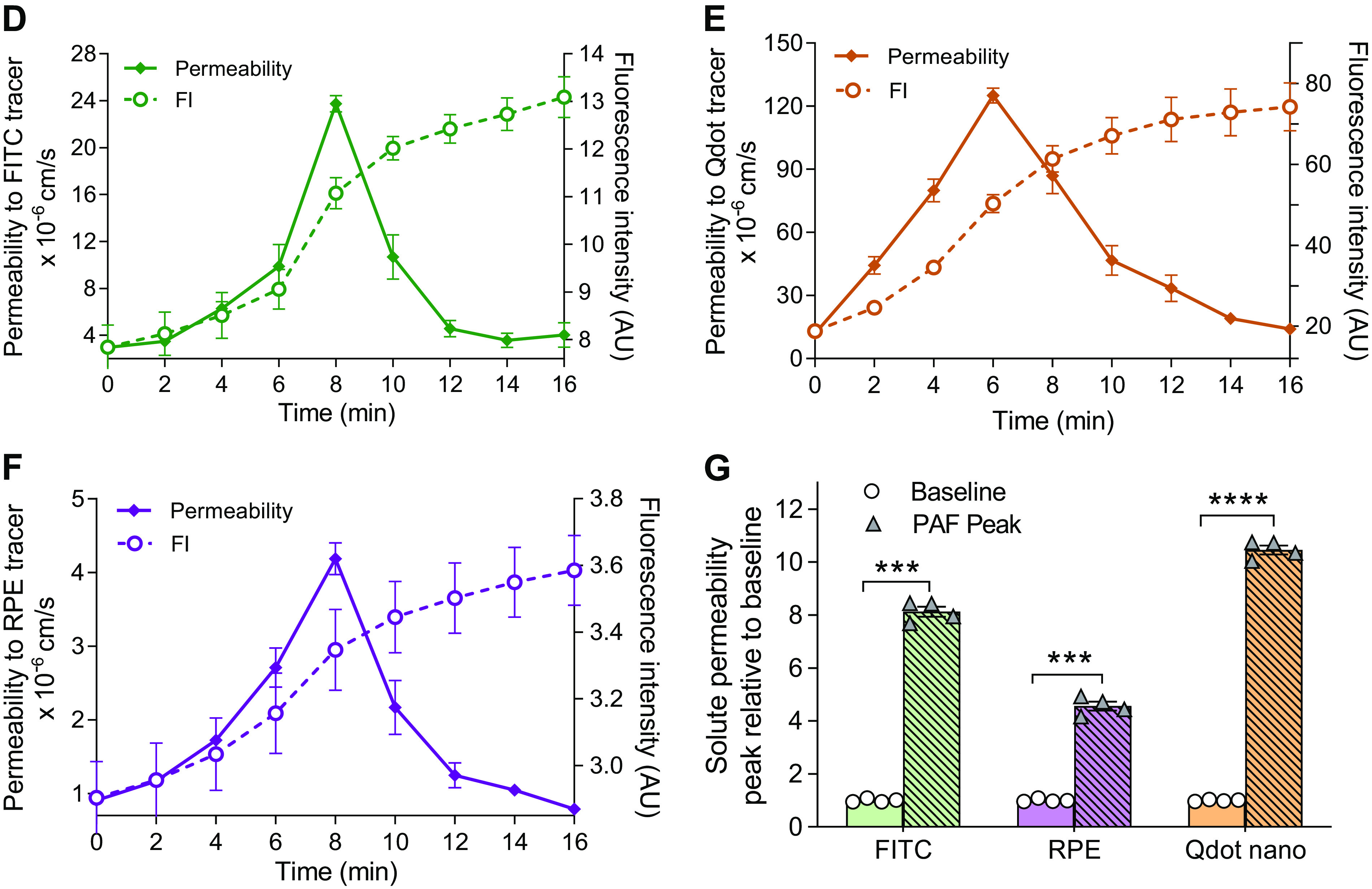
Permeability coefficient (*P*_s_) measurements in in vitro microfluidic microvessels under basal and stimulated conditions. *A*: summary results of baseline *P*_s_ to FITC-Avidin, R-Phycoerythrin (RPE)-Neutr-Avidin, and Qdot-Strept-Avidin (*n* = 8/group). *B*: confocal images showing the transport of FITC-Avidin, RPE-Neutr-Avidin, and Qdot-Strept-Avidin under baseline and platelet-activating factor (PAF 10 nM) stimulated conditions. The images illustrate the time-dependent accumulation of each avidin tracer across endothelium and stabilized on spot by substrate biotin. The spatial pattern of avidin accumulation outlines the transport pathways (paracellular vs. transcellular). *C*: schematic illustration of PAF-induced increases in avidin transport and the transport pathways correlating to the pattern of each avidin tracer accumulation shown in *B*. *D*–*F*: summary graph showing the overlay plot of the time-dependent cumulative fluorescence intensity (FI) profile of each avidin tracer and the calculated values of *P*_s_ to that tracer as a function of PAF perfusion time (*n* = 4/group). *G*: summary graph showing PAF-induced peak increases in *P*_s_ to each tracer relative to its baseline control (*n* = 4/group). Paired *t* test was used for statistical analysis. ***P < 0.0004 and ****P < 0.0001. AU, arbitrary unit. *n* is the number of devices used in each group.

The baseline permeability coefficients (*P*_s_) to fluorescence-labeled avidin tracers, FITC-Avidin, RPE-Neutr-Avidin, and Qdot-Strept-Avidin, were measured during perfusion of BSA-Ringer solution containing each of the tracers at a concentration of 0.09 µM in microvessel networks (1 tracer per device, *n* = 8 devices/group). The baseline *P*_s_ values of three tracers are summarized in Fig. 4*A.* The mean baseline *P*_s_ to FITC-Avidin (66–69 kDa, isoelectric point, pI, at 10.5) was 2.83 ± 0.06 × 10^−6^ cm/s, which falls into the range of the *P*_s_ to albumin (similar molecular mass to Avidin) measured in isolated rat skeletal muscle venules, and is ∼5 times higher than that measured in rat mesentery venules (26–28). RPE-Neutr-Avidin is a large fluorescent-labeled deglycosylated avidin with pI at 4.8 (RPE) and 6.5 (Neutr-Avidin), respectively. Its molecular mass is ∼4.5 times larger than FITC-Avidin. The *P*_s_ for this large RPE-Neutr-Avidin is 0.91 ± 0.01 × 10^−6^ cm/s, which is ∼30% of the *P*_s_ value of FITC-Avindin. Confocal images in Fig. 4*B* (*left* 3 columns) illustrate that a minimal or invisible amount of FITC-Avidin and RPE-Neutr-Avidin crossed endothelium under basal conditions. The streptavidin linked Qdot 800-conjugate is an even larger molecule with size ∼15 to 20 nm. However, these large molecules, likely due to the amphiphilic property of coated Qdot (29), enable to cross the cell membrane. The baseline images of Qdot-Strept-Avidin in Fig. 4*B* show a uniform, low level of Qdot-Strept-Avidin binding at substrate nonnuclear regions, indicating that these molecules crossed endothelium through transcellular pathways as opposed to paracellular pathways. The measured baseline *P*_s_ for Qdot-Strept-Avidin is 11.37 ± 0.30 × 10^−6^ cm/s, the highest value among the three tracers. More prominent distinctions of the tracer accumulation patterns are illustrated under stimulated conditions with increased permeability (results of the next section).

### Platelet-Activating Factor Increases Permeability Coefficient of the In Vitro Microvessels

PAF, an inflammatory mediator, has been demonstrated to cause transient increases in permeability and gap formation between ECs in intact rat mesenteric venules (30, 31). In this study, we used PAF to assess the changes in *P*_s_ and define the transport pathways in microfluidic microvessels. After measuring baseline *P*_s_ to an individual tracer, each device was perfused with PAF (10 nM) in the presence of the same concentration of tracer as that used for control. The confocal images were then collected every 2 min for 16 min during PAF perfusion (Fig. 4*B*). The changes in fluorescence intensity (FI) and the calculated *P*_s_ of each tracer as a function of PAF perfusion time are plotted in Fig. 4, *D*–*F* (*n* = 4 devices/group). The FI curve of each tracer represents the cumulative tracer molecules captured by biotin at substrate over time and the slope of the FI increase is the tracer accumulation rate (d*I*/d*t*), i.e., the solute flux. The plateau of the FI curve indicates the termination of the increased solute flux. The PAF-induced increases in *P*_s_ to each tracer derived from d*I*/d*t* are overlaid with the FI curve. A transient increase in *P*_s_ was observed in all three tracers. The increased *P*_s_ peaked 6–8 min after the start of PAF application and returned to control values in 14–16 min.

The *P*_s_ to FITC-Avidin increased from a mean baseline of 2.91 ± 0.1 × 10^−6^ cm/s to a peak of 23.60 ± 0.52 × 10^−6^ cm/s at 8 min of PAF perfusion, which is 8.13 ± 0.19 times that of the control (Fig. 4, *D* and *G*). The confocal images in Fig. 4*B* (*top* row) illustrate the spatial distribution of the crossed FITC-Avidin at substrate over a 16 min of PAF exposure. The crossed tracer molecules localized along all junctions between ECs in a relatively uniform pattern, indicating that all ECs reacted to PAF in a similar manner and the transport of FITC-Avidin was through intercellular gaps formed between ECs. The *P*_s_ to RPE-Neutr-Avidin increased from a mean base value of 0.90 ± 0.03 × 10^−6^ cm/s to a peak of 4.11 ± 0.09 × 10^−6^ cm/s, ∼4.56 times that of the control (Fig. 4, *E* and *G*). This larger RPE-Neutr-Avidin molecule was only detected at tricellular junctions, where three cells meet and often form larger gaps than those at bilateral junctions (Fig. 4*B*, *middle* row). These results indicate that the transport of both FITC-Avidin and RPE-Neutr-Avidin is through paracellular pathways and that the sizes of the gaps and the size of the solute determine the magnitude of the flux. In contrast to the spatial accumulation patterns of FITC-Avidin and RPE-Neutr-Avidin, the transport of Qdot-Strept-Avidin is notably through the whole cell layer, i.e., transcellular pathways (Fig. 4*B*, *bottom* row). The *P*_s_ to Qdot-Strept-Avidin increased from a baseline of 11.95 ± 0.19 × 10^−6^ cm/s to a peak of 125.02 ± 3.60 × 10^−6^ cm/s at 6 min of the PAF perfusion (10.46-fold increase, Fig. 4, *F* and *G*). The PAF-induced peak increases in *P*_s_ for each tracer relative to its baseline control are summarized in Fig. 4*G*. Figure 4*C* provides the schematic illustration of the transport pathways simulating the pattern of each tracer accumulation as shown in Fig. 4*B.*

### PAF-Induced Increases in EC [Ca^2+^]_i_ and Permeability Coefficient

**Figure 5.**
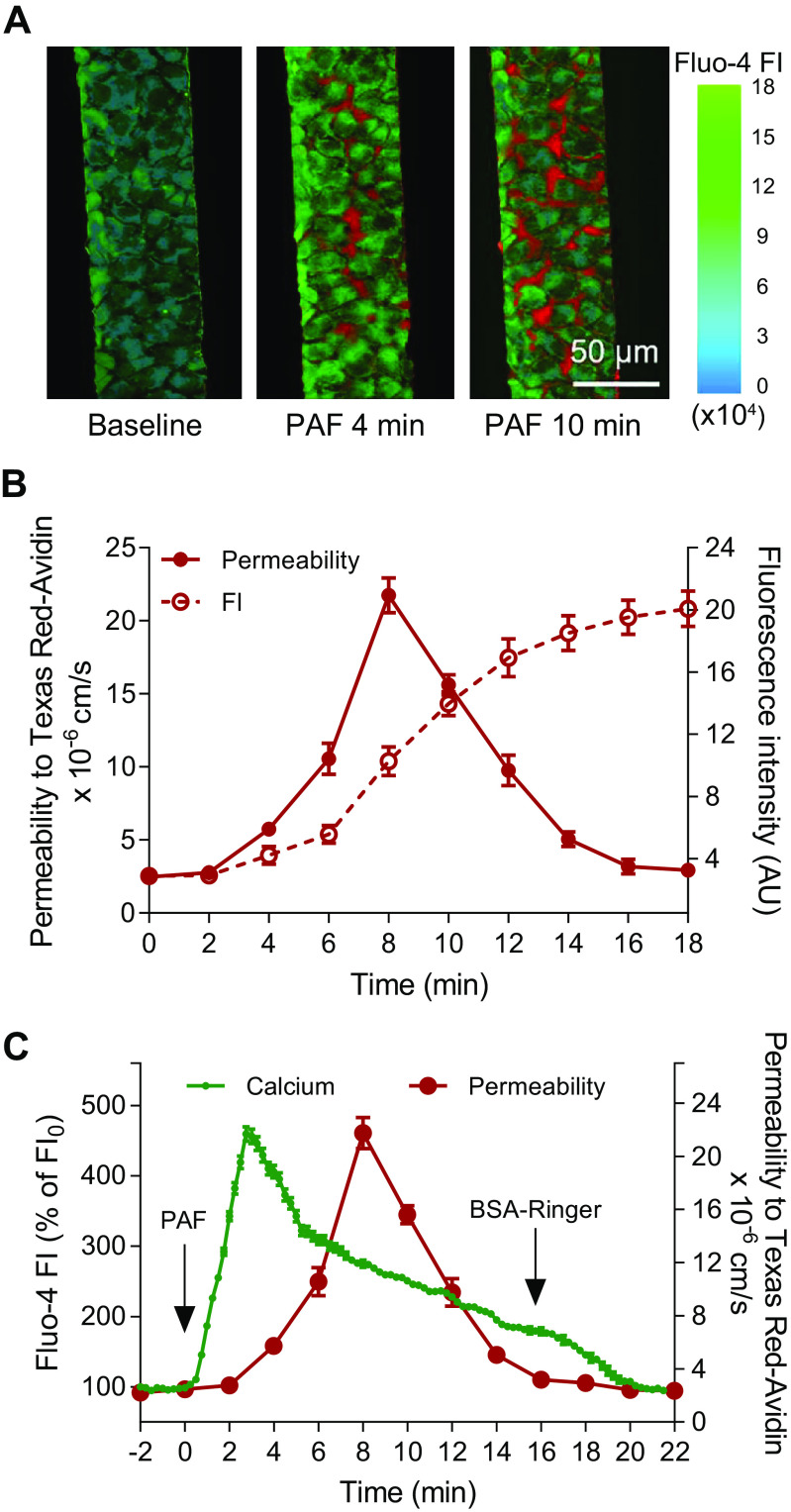
Platelet-activating factor (PAF)-induced increases in endothelial cell (EC) [Ca^2+^]_i_ and permeability coefficient (*P*_s_) to Texas Red-Avidin in microfluidic microvessels. *A*: confocal images showing the simultaneous changes in EC [Ca^2+^]_i_ and Texas Red-Avidin transport before and after the start of PAF perfusion for 4 and 10 min. Green channel shows PAF-induced changes in Fluo-4 fluorescence intensity (FI) representing the changes in EC [Ca^2+^]_i_ and the red channel shows PAF-induced Texas Red-Avidin accumulation at the substrate of the vessel wall. *B*: the overlay plot of the cumulative fluorescence intensity (FI) of Texas Red-Avidin and the calculated *P*_s_ as a function of PAF perfusion time (*n* = 4 devices per group). *C*: the superimposed plot of PAF-induced increases in EC [Ca^2+^]_i_ and *P*_s_ to Texas Red-Avidin as a function of time following PAF perfusion, illustrating their temporal relationship (*n* = 4 devices/group). AU, arbitrary unit.

Previous studies conducted in intact microvessels demonstrated that the agonist-induced increase in EC [Ca^2+^]_i_ is an initiation signal for EC gap formation and subsequent increases in microvessel permeability (22, 30, 32–35). However, EC [Ca^2+^]_i_ and permeability were measured in separate experiments. In this study, we are able to measure the changes in *P*_s_ and EC [Ca^2+^]_i_ simultaneously, which enables us to precisely define their temporal relation upon PAF application. EC [Ca^2+^]_i_ was measured with a fluorescent Ca^2+^ indicator, Fluo-4. After loading Fluo-4 am into ECs lining the microchannel networks, the free Fluo-4 AM in the vessel lumen was washed out with BSA-Ringer perfusion before collecting images for baseline and PAF-induced increases in EC [Ca^2+^]_i_. [Ca^2+^]_i_ images were collected at 15-s intervals. The changes in *P*_s_ were assessed with perfusion of Texas Red-Avidin in the same vessel segment where [Ca^2+^]_i_ images were collected. The images of Texas Red-Avidin accumulation were collected at 2-min intervals consistent with measurement of *P*_s_ alone. Figure 5*A* shows the representative images of Fluo-4 FI changes and the accumulation of Texas Red-Avidin before and after PAF exposure at selected time points. Figure 5*B* shows the overlay of the FI profile of Texas Red-Avidin and the *P*_s_ changes as a function of time following PAF (10 nM) application (*n* = 4 devices). *P*_s_ to Texas Red-Avidin increased from a mean base value of 2.46 ± 0.24 × 10^−6^ cm/s to a peak of 21.74 ± 1.19 × 10^−6^ cm/s 8 min after the start of PAF perfusion and declined close to the basal level at 16 min, a pattern and magnitude of *P*_s_ increase similar to that of FITC-Avidin (Fig. 4*D*). Figure 5*C* plots the time course of PAF-induced increases in EC [Ca^2+^]_i_ against that of the *P*_s_ (*n* = 4 devices). Results showed that EC [Ca^2+^]_i_ increased immediately following PAF application and reached a peak value at 4.65 ± 0.09 times that of the control in 3 min. However, *P*_s_ did not increase till ∼2 min after the start of PAF perfusion and reached its peak in 6 min, demonstrating a 5-min separation between the two peaks. The overlay plot (Fig. 5*C*) illustrates the temporal relationship between these two sequential events.

### Role of Glycocalyx in Microvessel Permeability

**Figure 6.**
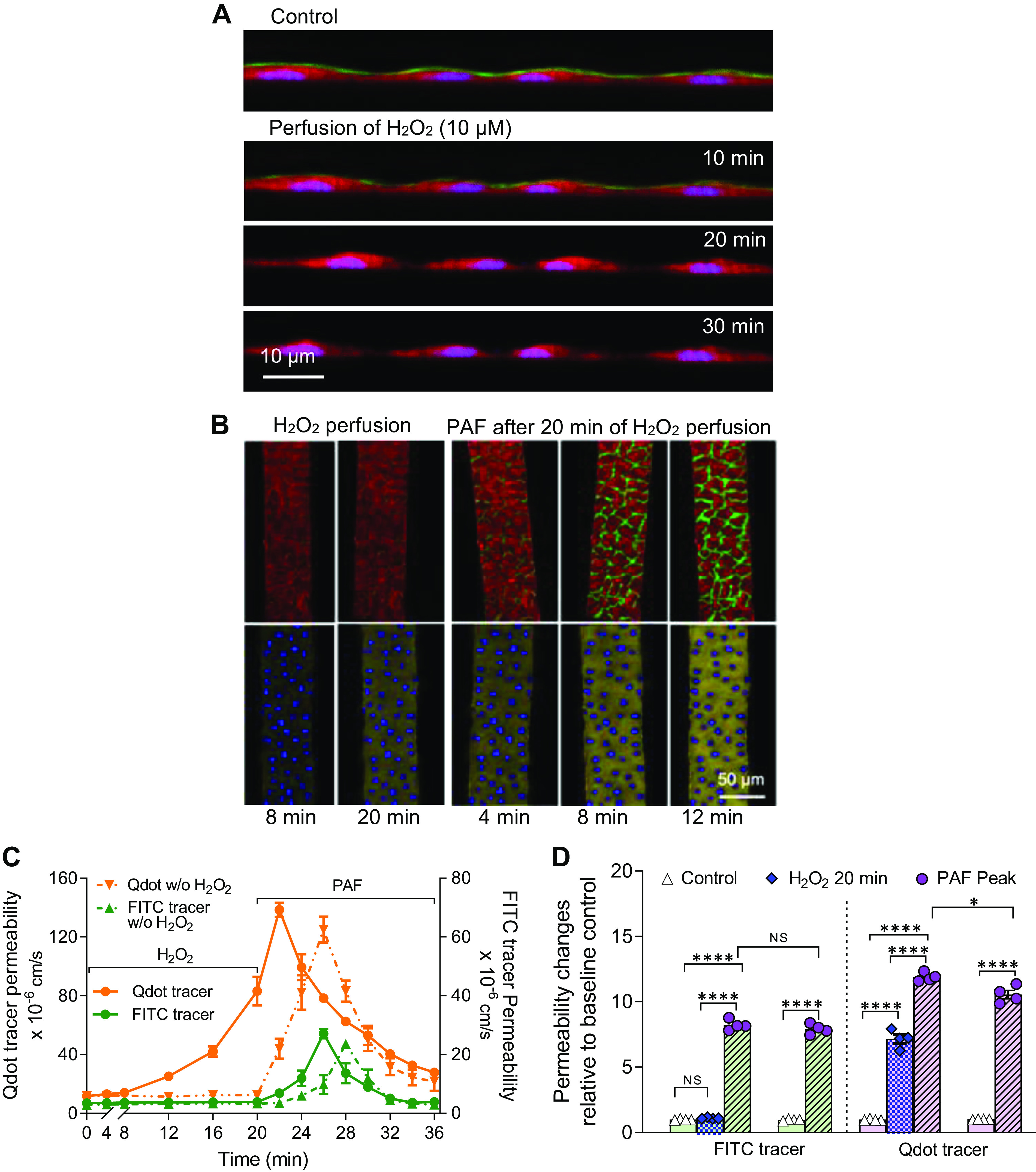
Glycocalyx and its role in basal and PAF-induced permeability increase in microfluidic microvessels. *A*: representative confocal images showing FITC-lectin-labeled glycocalyx (green) and Calcein Red-labeled endothelial cells at *X–Z* focal plane of the vessel wall. Microfluidic microvessels possess a well-formed glycocalyx layer (green) above endothelium under control conditions (*top* image). The *bottom* three confocal images show time-dependent degradation of glycocalyx during perfusion of H_2_O_2_ at 10 µM. A complete depletion of glycocalyx occurred at 20 min of H_2_O_2_ perfusion. *B*: confocal images demonstrating time-dependent accumulation of FITC-Avidin (green, *top* row) and Qdot-Strept-Avidin (yellow, *bottom* row) at endothelial cell (EC) substrate during H_2_O_2_ (10 µM) perfusion (*left* 2 columns) followed by the application of PAF (10 nM, *right* 3 columns). Calcein Red was used to outline ECs in images of the *top* row. DAPI (blue) was used to outline EC nuclei in images at the *bottom* row. *C*: superimposed time courses of the *P*_s_ changes during H_2_O_2_ perfusion and the application of PAF with and without the initial perfusion of H_2_O_2_. Orange curves are the time courses of *P*_s_ to Qdot-Strept-Avidin (*left y*-axis) and green curves are the time courses of *P*_s_ to FITC-Avidin (*right y*-axis), *n* = 4 devices/group. *D*: summary results of the *P*_s_ values under control, after glycocalyx degradation by H_2_O_2_ perfusion and PAF-induced peak increases in *P*_s_ in the presence and absence of glycocalyx. The *left* two columns with green color are *P*_s_ to FITC-Avidin and the *right* two columns with purple color are *P*_s_ to Qdot-Strept-Avidin. Each column data are paired experiments conducted in the same vessel segment (*n* = 4 devices/group). Paired *t* test was used for paired data comparisons and one-way ANOVA is used for between-group comparisons. NS *P* > 0.3, *P = 0.01, ****P < 0.00001. NS, not significant; PAF, platelet-activating factor.

Glycocalyx has been implicated to play an important role in microvessel barrier function (36, 37), and glycocalyx degradation was often associated with oxidative stress under disease conditions (38–43). Currently, the direct impact of degraded glycocalyx on microvessel permeability remains controversial (36, 44–46). Here, we first demonstrated that microvessels developed in microfluidics possess a well-formed layer of glycocalyx on the surface of endothelium. The control image in Fig. 6*A* illustrates FITC-lectin-labeled glycocalyx lining the vessel wall at an *X–Z* focal plane. The mean thickness of FITC-lectin-labeled glycocalyx was 422 ± 17 nm under control conditions (averaged from 5 devices and 5 regions per device), which fell into the thickness range of glycocalyx reported in mammalian capillaries (36, 38, 47).

To investigate the role of glycocalyx in microvessel permeability, we perfused the microvessels with a pathologically relevant concentration of H_2_O_2_ (10 µM) and illustrated a time-dependent glycocalyx degradation and concomitant changes in *P*_s_ to different solutes with real-time confocal imaging. The time-dependent changes in glycocalyx during H_2_O_2_ perfusion are illustrated in Fig. 6*A* with simultaneously labeled ECs (Calcein Red) and glycocalyx (FITC-lectin) at a cross-sectional view (*X–Z* focal plane). Images demonstrated that 20 min of H_2_O_2_ perfusion at 10 µM caused a complete depletion of glycocalyx. *P*_s_ to Qdot-Strept-Avidin and FITC-Avidin were measured before and during H_2_O_2_ perfusion. Images in Fig. 6*B* (*left* 2 columns) and Fig. 6*C* demonstrate that H_2_O_2_-induced depletion of glycocalyx did not cause significant changes in *P*_s_ to FITC-Avidin but increased *P*_s_ to Qdot-Strept-Avidin through transcellular pathways. *P*_s_ to Qdot-Strept-Avidin started to increase at 8 min of H_2_O_2_ perfusion, correlating to a partial glycocalyx degradation shown in Fig. 6*A.* At 20 min of H_2_O_2_ perfusion, *P*_s_ increased from a baseline of 11.53 ± 0.34 × 10^−6^ cm/s to 83.45 ± 3.98 × 10^−6^ cm/s, 7.24 ± 0.26 times that of the control. In contrast, *P*_s_ to FITC-Avidin showed no significant increase after 20 min of H_2_O_2_ perfusion and the images in Fig. 6*B* confirmed no significant accumulation of FITC-Avidin at EC junctions, indicating that H_2_O_2_ perfusion for 20 min with depleted glycocalyx did not affect EC junctions.

We then examined the effect of impaired glycocalyx on PAF-induced permeability increases. PAF was applied to vessel lumen after 20 min of H_2_O_2_ perfusion in each device. Upon PAF application, *P*_s_ to Qdot-Strept-Avidin further increased to a peak value of 138.58 ± 1.95 × 10^−6^ cm/s in 2 min. The mean peak value was slightly higher (*P* = 0.01) than that measured with intact glycocalyx, but the time to peak was 4 min faster than that of PAF application alone. *P*_s_ to FITC-Avidin increased from 3.77 ± 0.11 × 10^−6^ cm/s (after H_2_O_2_ perfusion for 20 min) to a peak value of 27.04 ± 0.67 × 10^−6^ cm/s 6 min after the start of PAF perfusion. The peak values of *P*_s_ to FITC-Avidin were not significantly different from vessels with intact glycocalyx, but the time to peak was 2 min faster with depleted glycocalyx. The *P*_s_ increases in the presence and absence of glycocalyx are shown in Fig. 6*C*, and the results are summarized in Fig. 6*D* (*n* = 4 devices/group).

## DISCUSSION

Our study introduces a novel method that allows the microvessel permeability coefficient (*P*_s_) to be measured in nonpermeable microfluidic platforms using avidin-biotin technology. This approach overcomes the limitations of nonpermeable microfluidic platforms and permits not only quantitative assessments of permeability properties of the microvessel wall but also spatial illustration of solute transport pathways. Using this newly developed approach, our study demonstrated that microvessels developed in a PDMS-based chip platform structurally possess in vivo-like EC junctions and glycocalyx surface layer and functionally recapitulate the basal barrier properties and *P*_s_ and EC [Ca^2+^]_i_ responses to inflammatory stimuli observed in intact microvessels. Permeability measurement is a critical assessment of the barrier function of microvessel wall, which has not been easily implanted in most of the microvessel-on-a chip models. This newly developed permeability assessment approach overcomes the major limitation of nonpermeable microfluidic platforms and offers a well-characterized in vitro microvessel model for a wide range of applications in human disease studies and pharmaceutical screening.

### Permeability Assessments in Microvessels-on-Chip Platforms

Microvessels in vivo have multiple layers of barrier contributing to the selectivity and quantity of fluid and solute transport, which are the surface layer of glycocalyx, endothelium, basement membrane, and surrounding perivascular cells such as pericytes or smooth muscle cells. Under basal conditions, glycocalyx and endothelium are the main barriers excluding macromolecules across the vascular wall (1). Under chronic inflammatory conditions, when the glycocalyx and endothelial barriers are compromised, the basement membrane and perivascular cells could stabilize the vascular wall, serving as additional barriers to lessen the extent of increased permeability (31). Our single-channel-patterned microvessel network platform is composed of a single layer of endothelial cell-formed microvessels, lacking surrounding perivascular cells and in vivo-like extracellular matrices. However, this study demonstrates that microfluidic microvessels formed by a single layer of endothelial cells possess in vivo-like glycocalyx and junctional proteins and recapitulate the basal barrier function and EC responses to inflammatory stimuli of intact microvessels in vivo. These results indicate that the in vivo-like endothelial junctions and glycocalyx layer developed under continuous flow in microfluidic devices are the key components contributing to their barrier function, making distinctions from the proinflammatory phenotype often observed in endothelial monolayers cultured under static conditions (2, 36).

The hydrogel-coated microchannels or self-assembled microvascular networks within hydrogel matrices better represent the in vivo extracellular environment than ECs grown in collagen or fibronectin-coated none-permeable microfluidic channels. Those chip platforms make them well suited for investigations that focus on the influence of perivascular environment in microvascular development. However, the hydrogel-based platforms add additional complexity and variables for permeability assessment, such as composition-dependent diffusive properties of the hydrogel and the inconsistent patterns of self-assembled microvessel networks (21, 48). These variables create challenges for applications that require a reproducible in vitro microvessel model with controlled vascular patterns and flow dynamics, as well as consistent baseline permeability to serve as a standardized control before further experimentations. In addition, the hydrogel-based platforms often rely on electron microscopy or additional intracellular staining to define transport pathways. In contrast, the PDMS-based microfluidic devices used in our study are easy to create desired microchannel patterns with predictable flow dynamics under defined flow conditions. Importantly, the in vitro microvascular networks developed in such devices are highly reproducible. It also allows smaller channel diameters with longer term stability of microvessels than those developed in three-dimensional tissue scaffolds (7). Within microvasculature, postcapillary venules are the main sites of leakages during inflammation (1, 2). Studies conducted in individually perfused rat mesenteric venules showed that the diameters of those venules are in a range of 35–50 µm (22, 30, 34). The smaller microchannels used for this study have diameter of 60 µm, which is very close to in vivo venular diameters we have studied previously. Despite these unique features and advantages of PDMS-based microfluidic devices over other platforms, the major limitation of such chip platforms is the nonpermeable microchannels that prevent the assessments of permeability. Our newly developed in vitro microvessel model overcomes this limitation and presents an easily executed approach that enables the quantification of permeability coefficients to be conducted in nonpermeable microfluidic devices, as well as the direct visualization of solute transport pathways. This model advances the microfluidic technology and opens up a wide range of translational applications such as investigations of patient blood interactions with human microvascular ECs to detect pathological factors that increase permeability, to identify cellular and molecular signaling pathways that regulate permeability, and to conduct mechanistic investigations for drug delivery and screening processes.

Reviewing some of the microvessel permeability measurements conducted in intact and isolated microvessels (Table 1), the values vary based on the solute size and charge, as well as the selectivity or structural variations of the microvessel wall among species, tissue origin, and vessel type (23, 26–28, 49, 50, 52–54). Based on studies by Sarelius et al. (26), the *P*_s_ of albumin in venules is ∼3–4 times higher than that of arterioles measured in the same tissue, reflecting the differences in vessel wall cell composition and structure. The venular *P*_s_ in muscle microvessels in general is higher than those in mesenteries, reflective of their differences in organ or tissue origins (26–28, 51, 52). In this study, the microfluidic microvessels were developed from rat dermal microvessel ECs. Our measured basal *P*_s_ values to FITC-Avidin and Texas Red-Avidin (66–69 kDa) that have similar molecular mass to albumin (55) but carry positive charges (pI at 10.5) are in the range of *P*_s_ to albumin measured in intact muscle venules (26). RPE-Neutr-Avidin (300 kDa), a large fluorescence molecule conjugated deglycosylated avidin with increased mass and more neutral isoelectric points (4.8 for RPE and 6.5 for Neutr-Avidin) had ∼30% of the *P*_s_ value to FITC-Avidin, demonstrating the solute size selectivity of the microvessel wall. However, Qdot-Strept-Avidin (15–20 nm), the largest tracer we used for this study, was able to cross cell membrane and served as a useful tracer to assess solute transport through transcellular pathways. One advantage of using the nonpermeable chip platform is that the detected solute flux at biotin substrate is the result of net diffusion without convective (solvent drag) component and the influence of diffusion variations of the surrounding tissue.

**Table 1.** Solute permeability values measured in intact vessel and microfluidic microvessels

Species/Organ	Vessel Type	Solute	Baseline *P*_s_ 10^−6^ cm/s	Stimulation	*P*_s_ Peak 10^−6^ cm/s	Reference
Frog mesentery	Venules	α-Lactalbumin	2.1			(23)
		Albumin	1.41 ± 0.67			(27)
		LDL	0.78 ± 0.14	Ca Ionophore A23187 (5 µM)	9.62 ± 1.56	(49)
		Dextran-20	0.32 ± 0.29		11.42 ± 1.57	
Hamster mesentery	Venules	LDL	0.27	Histamine (100 µM)	2.32	(50)
Swine coronary	Venules	Albumin	3.74 ± 0.45	Histamine (10 µM)	10.24 ± 1.97	(51)
			1.48 ± 0.62			(27)
Mouse cremaster	Arterioles	Albumin	0.99 ± 0.11			(26)
	Venules		4.44 ± 0.79		
Rat skeletal muscle	Arterioles	Albumin	0.84 ± 0.13		
	Venules		2.50 ± 0.37		
Rat mesentery	Venules	α-Lactalbumin	5.0 ± 0.4	TNF-α	5.2 ± 0.6	(52)
		α-Lactalbumin	4.4 ± 0.5	VEGF (1 nM)	15 ± 1.9	(28)
		Albumin	0.49 ± 0.03		3.6 ± 0.32
			0.56 ± 0.20	
Rat dermal microvess-el ECs	In vitro microvessels in microfluidics	FITC-Avidin	2.91 ± 0.1	PAF (10 nM)	23.60 ± 0.52	This study
		Texas Red-Avidin	2.46 ± 0.24		21.74 ± 1.19	
		RPE-Neutr-Avidin	0.90 ± 0.03		4.11 ± 0.09	
		Qdot-Strept-Avidin	11.95 ± 0.19		125.02 ± 3.60	

ECs, endothelial cell; PAF, platelet-activating factor; RPE, R-Phycoerythrin.

### Solute Transport through Paracellular versus Transcellular Pathways

Cumulative research evidence indicated that solute and fluid transport across microvascular endothelium involve both transcellular and paracellular pathways (1). In continuous endothelium, the transcellular transport of certain small molecules is receptor-mediated and macromolecules such as albumin are transported by vesicles or cluster of caveolae and vesiculo-vacuolar organelles (VVOs, vesicle formed channels across ECs) (1, 56–59). Paracellular transport is regulated by the junctions between ECs and the openings or gaps formed between ECs upon exposure to inflammatory mediators have been considered as the primary pathways for increased permeability, especially in postcapillary venules (1, 30, 60, 61).

Paracellular transport under stimulated or inflammatory conditions has been evidenced by the junctional accumulations of carbon or fluorescent microspheres and by outlining EC borders with silver nitrate deposition through microscopic images and electron micrographs (30, 60–62). Studies showed that inflammatory mediators such as histamine, substance P, or PAF all cause transient increases in permeability. Importantly, the magnitude of increased permeability is correlated with the number and size of formed gaps, and the permeability recovery phase is associated with gap closure (30, 63). Our microfluidic in vitro microvessel studies resembled those findings. We observed transient increases in permeability upon PAF stimulation with peak occurred at 6–8 min after the start of PAF perfusion. The declining phase of increased permeability was correlated with the plateau of solute accumulation (fluorescence intensity) indicating no further increased solute flux. However, it is reasonable to challenge that this plateau of fluorescence intensity was the result of binding saturation due to insufficient available biotin for crossed avidin molecules. Our biotin-binding capacity test shown in Fig. 2*B* demonstrates that perfusion of 0.09 µM FITC-Avidin to the biotin-coated microchannels increased FITC intensity at coating layer in a time-dependent manner and reached 224 (AU) after 90 min of perfusion, whereas the maximum cumulated mean FI value in Fig. 4*D* (FITC-Avidin) was ∼13 (AU) and the maximum FI values of biotin-avidin at cell junctional regions at the end of 16 min of PAF perfusion were in a range of 17–38 (AU). These results provide experimental evidence that the 1:10,000 avidin to biotin concentration ratio used in our experimental setting provided sufficient biotin to catch all crossed avidin molecules during the experiments. The transient increases in *P*_s_ during PAF perfusion were not due to the saturated avidin binding to the available biotin. Importantly, the transient nature of the permeability responses to PAF and many other inflammatory mediators such as bradykinin, ATP, etc., has been well documented (22, 30, 32–35). Our results are consistent to those studies.

Because of the high binding affinity between avidin and biotin, all crossed avidin molecules were registered on the spot of biotinylated substrate and thereby spatially outlined the sites of leakage. The traversed FITC-Avidin and RPE-Avidin molecules were all deposited at the EC junctional area of the substrate, indicating that they crossed endothelium via paracellular pathways. With identical stimulation and consistent EC responses to PAF, our results demonstrated that the magnitude of the solute flux depends on the size of both the solute and the EC gaps. The peak *P*_s_ to RPE-Neutr-Avidin (larger molecule) is only about half of the peak *P*_s_ to FITC-Avidin. The crossed FITC-Avidin located along all junctions between ECs, whereas RPE-Neutr-Avidin (4.5 times the size of FITC-Avidin) was only detected at tricellular junctions where there were larger gaps than those at bilateral junctions (Fig. 4*B*, *middle* row), suggesting that the size of RPE-Neutr-Avidin was larger than majority of EC bilateral gaps. In contrast to the pattern of FITC-Avidin and RPE-Neutr-Avidin deposition outlining EC junctions, indicative of paracellular transport, Qdot-Strept-Avidin were deposited at the entire nonnuclear region of the substrate under both basal and stimulated conditions, illustrating their transcellular route across endothelium. This observation is consistent with another study using cultured EC monolayers (20). The mechanism whereby Qdot-Strept-Avidin is able to penetrate plasma membrane and cross endothelium remains unclear. Variable Qdot products have been used as live cell trackers and the uptake of Qdot nanocrystals is considered passive and nonreceptor mediated and can be incorporated in cytoplasmic vesicles (64, 65). Currently, whether transcellular pathways contribute to the agonist-induced increases in macromolecule transport remain under debate (1, 63). Increased frequency of vacuoles and VVOs has been observed in inflammatory mediator-stimulated endothelium of microvessels (66, 67). Our results showed that *P*_s_ to Qdot-Strept-Avidin increased over 10-fold in response to PAF, which further support the role of transcellular transport in increased permeability.

### Temporal Relationship between PAF-Induced Increases in EC [Ca^2+^]_i_ and Microvessel Permeability

Studies in individually perfused intact microvessels indicate that inflammatory mediator-induced increases in EC [Ca^2+^]_i_ are required to increase microvessel permeability and that the peak level of EC [Ca^2+^]_i_ determines the extent of gap formation and increased microvessel permeability (22, 30, 32–35). However, in previous studies, the changes in EC [Ca^2+^]_i_ and permeability were measured separately in different vessels, and therefore, their temporal relationship could not be well defined. This newly developed permeability assessment in microfluidic microvessels permits simultaneous measurements of EC [Ca^2+^]_i_ and *P*_s_, which precisely defines their temporal relationship during PAF exposure. Our results demonstrated an immediate increase in EC [Ca^2+^]_i_ but delayed *P*_s_ response upon PAF exposure. The immediate increase in EC [Ca^2+^]_i_ in response to PAF was consistent with that observed in ATP-exposed microfluidic microvessels (6). Previous studies in intact microvessels also demonstrated that agonist-induced calcium release from internal stores alone (∼20% to 30% of the [Ca^2+^]_i_ peak value) in the absence of extracellular Ca^2+^ or restricting Ca^2+^ influx to a very low level abolished the permeability increases, indicating that an above a threshold EC [Ca^2+^]_i_ level is required to sufficiently increase permeability (32, 33). Linking these observations to the 2-min delay of *P*_s_ response observed in this study, we predict that the delayed *P*_s_ increase may reflect the time needed for EC [Ca^2+^]_i_ increased to a level sufficient to initiate the calcium-dependent interaction of actin/myosin in ECs, followed by the activation of cytoskeletal contractile machinery to form gaps between ECs, as well as the start of increasing solute transport across the endothelium. The 5-min separation between the two peak values indicates that the maximum cellular actions and solute transport in response to the peak of EC [Ca^2+^]_i_ has a further delay by ∼3 min.

### Role of Glycocalyx Layer in Vascular Permeability

Glycocalyx that lines the luminal surface of entire vasculature has been recognized to play important roles in vascular barrier homeostasis, such as the selectivity for solute transport, the rate of solute penetration, vascular permeability, mechanotransduction, leukocyte adhesion, etc. (1, 36, 37). The degradation and shedding of glycocalyx have been found under a variety of pathological conditions (38–43). However, the direct impact of degraded glycocalyx on microvessel permeability remains controversial (36, 44–46). Enzymatic partial digestion of a thinner layer of glycocalyx (150 nm) in frog capillaries by pronase has shown to increase hydraulic conductivity (Lp) by about twofold without changing the intercellular cleft (68). Although others showed that glycocalyx shedding-associated alterations of vascular permeability in rat vessels depended on the composition of perfusion fluid, suggesting that glycocalyx shedding itself does not alter permeability (44, 46). In this study, we used a pathologically relevant concentration of H_2_O_2_ (10 µM) to degrade glycocalyx and demonstrated the real-time changes in glycocalyx and their corresponding changes in permeability. It is important to point out that H_2_O_2_ at 10 µM is a concentration close to the human plasma levels of H_2_O_2_ under pathological conditions (69, 70). We consider the plasma H_2_O_2_ concentration to be the net balance of the constant production of reactive oxygen species and their reactions with plasma enzymes such as SOD and catalase in the vasculature, representing the effective level of peroxide that affects endothelial cells lining the vascular walls. An important feature of the use of individually perfused microvessels in vivo or microvessels-on-a-chip in vitro for the H_2_O_2_ studies is the constantly refreshed perfusate in the vessel lumen, which exposes the endothelium with a relatively constant concentration of H_2_O_2_, closely simulating the in vivo situation. This constantly replenished H_2_O_2_ in the perfusate makes a fundamental difference from the one-time addition of reactive reagents to the tissue or cell culture medium that results in an immediate reaction of the reactive agents with the cells and other components in the medium and lost its activity quickly. In those studies, usually hundreds of μmol/L or mmol/L concentrations of H_2_O_2_ were required to show some of the responses (71–74). Our previous study demonstrated that perfusion of rat mesenteric microvessels with H_2_O_2_ at 10 µM had no immediate effect on hydraulic conductivity, Lp, and EC [Ca^2+^]_i_, and that the increases in Lp occurred after 1 h of H_2_O_2_ perfusion (75). In this study, we demonstrate that H_2_O_2_ (10 µM) perfusion depleted glycocalyx in ∼20 min. The H_2_O_2_-induced quick degradation of glycocalyx and its delayed Lp effect implies that glycocalyx may not have an important role in the maintenance of basal Lp in rat microvessels. Here, the role of glycocalyx in microvessel barrier function is further evaluated by measuring *P*_s_ to solutes with different physiochemical properties under both basal and stimulated conditions before and during glycocalyx degradation with H_2_O_2_. Results demonstrated that depletion of glycocalyx did not alter basal *P*_s_ to FITC-Avidin, endothelial junctions, and PAF-induced peak *P*_s_ increase, but shortened the time to PAF-induced *P*_s_ peak by 2 min when compared with that with intact glycocalyx. The reduced time to PAF-induced peak indicates that glycocalyx restricts the rate of solute penetration but does not alter PAF-induced intercellular gap formation and the maximum amount of solute across endothelium through paracellular pathways. In contrast, glycocalyx depletion not only increased basal *P*_s_ to Qdot-Strept-Avidin through transcellular pathways by sevenfold but also further increased its PAF-induced peak value and shortened the time to peak by 4 min. The latter observations indicate that glycocalyx plays important roles in restriction of selective molecules and their transport through transcellular pathways. The depletion of glycocalyx, although not affecting EC junctions and paracellular transport, increases transcellular transport under both basal and stimulated conditions. Currently, there has been no in vivo information about the real-time effect of H_2_O_2_ on glycocalyx shedding. An intravital microscopic study in hamster cremaster vessels demonstrated that a bolus intravenous injection of Ox-LDL transiently diminished the EC surface layer by 60% within 25 min by observing the changes in distance between erythrocytes and the capillary EC surface (76), which suggested that oxidative stress can cause the shedding of glycocalyx layer quickly in vivo.

### Summary

Our study demonstrated that microvessels developed under continuous flow conditions not only developed in vivo-like endothelial junctions but also a well-formed endothelial surface layer of glycocalyx. Importantly, the application of avidin-biotin technology to in vitro microfluidic microvessels overcomes the major limitations of microvessels developed in nonpermeable microfluidics. The permeability coefficient measurements using this newly developed technique further demonstrated that these in vitro microvessels recapitulate the barrier properties and the *P*_s_ and EC [Ca^2+^]_i_ responses to inflammatory stimuli observed in intact microvessels. Our study further demonstrated that the simultaneous measurements of *P*_s_, EC [Ca^2+^]_i_, as well as the real-time illustration of the alterations of EC junctions and glycocalyx in microfluidic microvessel networks would further advance microfluidics for blood research, especially for investigations of patient-specific blood component interactions with human endothelial cell-developed microvessels, benefiting both basic science and clinical applications.

## GRANTS

This work was supported by the National Heart, Lung, and Blood Institute Grants HL130363 and HL144620.

## DISCLOSURES

No conflicts of interest, financial or otherwise, are declared by the authors.

## AUTHOR CONTRIBUTIONS

F.G., H.S., and P.H. conceived and designed research; F.G., H.S., and X.L. performed experiments; F.G., H.S., X.L., and P.H. analyzed data; H.S. and P.H. interpreted results of experiments; F.G., H.S., X.L., and P.H. prepared figures; P.H. drafted manuscript; H.S., X.L., and P.H. edited and revised manuscript; F.G., H.S., X.L., and P.H. approved final version of manuscript.

**Table A1.** Excitation/emission wavelengths used for confocal images with fluorescence labeling

Tracer	Excitation Laser, nm	Emission Setting, nm
FITC-Avidin	488	510–530
RPE-NeutrAvidin	552	570–590
Qdot-streptavidin	638	660–690
Fluo-4 AM	488	510–530
Texas Red-Avidin	638	650–680
DAPI	405	440–460
Calcein Red	552	570–590
FITC-lectin	488	510–530

RPE, R-Phycoerythrin.

**Table A2.** Molecular mass and isoelectric point of the proteins used for tracers and permeability measurements

Protein	Molecular Mass	Size, nm	Isoelectric Point
Avidin	67–68 kDa		10.5
Neutravidin	60 kDa		6.3
Streptavidin	53 kDa		6.8–7.5
R-Phycoerythrin	240 kDa		4.8
FITC	589 mol wt		
Texas red	625 mol wt		
Albumin	66 kDa	3.8	4.7–5.6
α-Lactalbumin	14 kDa	2.01	4.6–5.2
LDL	2,300,000 kDa	19–28	
Qdot nanocrystals	>500 kDa	10–20	
Qdot-streptavidin	5–10 Streptavidins per Qdot using polyethylene glycol linker	15–20	

## APPENDIX

The excitation and emission laser wavelength settings used for each fluorescent tracer are listed in Table A1, and the physiochemical properties of the tracers are outlined in Table A2.
